# Intestinal Alkaline Phosphatase Expression in Response to *Escherichia coli* Infection in Nursery Pigs

**DOI:** 10.3390/ani15152179

**Published:** 2025-07-24

**Authors:** Sireethon Maksin, Attapon Kamlangdee, Alongkot Boonsoongnern, Prapassorn Boonsoongnern

**Affiliations:** 1 Program of Animal Health and Biomedical Sciences, Faculty of Veterinary Medicine, Kasetsart University, Bangkok 10900, Thailand; sireethon.ma@ku.th; 2 AK Sci and Consulting, Pathum Thani 12000, Thailand; autpon@gmail.com; 3 Department of Farm Resources and Production Medicine, Faculty of Veterinary Medicine, Kasetsart University, Nakhon Pathom 73140, Thailand; alongkot.b@ku.th; 4 Department of Anatomy, Faculty of Veterinary Medicine, Kasetsart University, Bangkok 10900, Thailand

**Keywords:** *Escherichia coli*, gut health, intestinal alkaline phosphatase, nursery pigs

## Abstract

Intestinal alkaline phosphatase (IAP) is a brush border enzyme secreted by enterocytes, playing a crucial role in maintaining gut mucosal defense. This study investigated the expression dynamics of IAP in the small intestine of pigs challenged with *Escherichia coli (E. coli)* K88, compared to healthy controls. Five-week-old pigs (n = 8) were orally administered *E. coli* K88 at a concentration of 2 × 10^8^ CFU/mL, with a dose of 2 mL per pig at 0 and 24 h. Five days post-challenge, tissue samples from the duodenum, jejunum, and ileum were collected for mucosal morphometric analysis and evaluation of IAP expression via immunohistochemistry, Western blotting, and real-time PCR. The results revealed the presence of IAP on the apical surface of villi throughout the small intestine, along with significantly upregulated IAP expression in *E. coli*-challenged pigs compared to controls. These findings suggest that Gram-negative bacteria such as *E. coli* can induce IAP expression, likely through lipopolysaccharide (LPS) stimulation, thereby enhancing its enzymatic activity as part of the intestinal defense mechanism. This study provides insight into the protective role of IAP and highlights its potential as a biomarker for assessing gut health and diagnosing enteric infections in animals.

## Simple Summary

Intestinal alkaline phosphatase (IAP) is an enzyme produced by cells lining the small intestine that plays a key role in maintaining gut health. In this study, we investigated how IAP responds in young pigs infected with *Escherichia coli* (*E. coli*) K88, a bacterium known to cause diarrhea. After exposure to *E. coli*, we found that IAP levels were significantly increased in the small intestine, particularly on the surface of the intestinal villi. This increase was observed using laboratory techniques such as immunohistochemistry and molecular analysis. The results suggest that IAP is activated in response to bacterial infection and may serve as a natural defense mechanism against harmful substances such as lipopolysaccharides (LPSs) from bacteria. Our findings highlight the potential use of IAP as a marker for gut health and a tool for detecting intestinal infections in animals.

## 1. Introduction

Piglet weaning is a critical stage of postnatal growth in a commercial pig production system and is often associated with gastrointestinal infections [1]. In particular, early weaning can have negative consequences, including increased susceptibility to stress (weaning stress), which can affect the growth performance and intestinal health of piglets [2,3]. Weaning stress can lead to diarrhea in piglets, increasing their susceptibility to pathogenic bacterial infections [4]. Post-weaning diarrhea (PWD) in piglets is a multifactorial disease associated with enterotoxigenic *Escherichia coli* (ETEC) strains [5]. It typically appears during the first two weeks after weaning. ETEC strains expressing F4 and F18 fimbriae are the primary pathogens responsible for PWD in piglets [6]. They impair gut health by adhering to intestinal enterocytes via fimbriae. The presence of fimbrial adhesins mediates attachment to porcine enterocytes, whereas enterotoxins disrupt fluid homeostasis in the small intestine, leading to watery diarrhea [7]. Moreover, ETEC infection promotes inflammation, as indicated by the expression of pro-inflammatory cytokines (IL-1β, IL-6, IL-17, IL-18, and TNF-α) in the jejunum [7]. PWD contributes to significant economic losses globally in swine production due to increased morbidity and mortality and decreased growth rates [5,8,9].

Intestinal alkaline phosphatase (IAP) is a small intestinal brush border enzyme secreted by enterocytes, playing a vital role as a gut mucosal defense factor [10,11]. IAP is expressed and secreted by intestinal epithelial cells, remaining active within both the mucosal membrane and the intestinal lumen [12]. IAP is essential for inactivating lipopolysaccharide (LPS) through dephosphorylation of its lipid A moiety [13]. In wild-type (WT) mice under normal conditions, LPS that crosses the intestinal brush border is typically in a dephosphorylated (inactive) form due to exposure to IAP. In contrast, IAP-knockout (KO) mice lack the ability to detoxify LPS in this manner [14]. In addition to investigating the effect of IAP in reducing the toxification of LPS, rats were orally exposed to LPS and L-phenylalanine, an IAP inhibitor, administered simultaneously with LPS. As a result, serum LPS levels increased twofold [15]. It has been reported that eight-week-old pigs were given calf intestinal alkaline phosphatase before LPS administration to neutralize and detoxify LPS [16].

Due to the properties of IAP in detoxifying LPS, this study aimed to reveal the relationship between IAP expression in the small intestine of pigs challenged with *E. coli* K88 compared to normal pigs, as this has become a key diagnostic or therapeutic marker for gut health in pigs.

## 2. Materials and Methods

### 2.1. Animals and Experimental Design

Sixteen growing pigs (5 weeks old) were obtained from Charoen Pokphand Foods Public Company Limited (CPF, Thailand) and housed at the farm animal demonstration and research facility of the Faculty of Veterinary Medicine, Kasetsart University. The animals were randomly assigned to two groups (n = 8 per group): a negative (control) group and a positive (treatment) group. The positive group was orally challenged with *Escherichia coli* (*E. coli*) strain K88 at a concentration of 2 × 10^8^ CFU/mL, administered at a dosage of 2 mL per pig at 0 and 24 h. The pigs were monitored daily, and fecal consistency was scored using a five-point scale based on the physical characteristics of the feces: 1 = firm and shaped, 2 = moist and shaped, 3 = loose, 4 = homogeneous watery, and 5 = watery with separated components. Scores ≥ 3 were operationally defined as indicative of diarrhea in this study.

At the end of the experiment (5 days post-challenge), all pigs were humanely euthanized via intravenous injection of sodium thiopental (100 mg/kg body weight). Intestinal tissue samples were collected from three segments—the duodenum, jejunum, and ileum—for further histological and molecular analyses. All animal procedures were reviewed and approved by the Animal Care and Use Committee of the Faculty of Veterinary Medicine, Kasetsart University, Thailand (ACKU64-VET-064).

### 2.2. Morphometric Analysis of the Small Intestinal Mucosa

Segments of the small intestine were fixed in 10% neutral-buffered formalin and embedded in paraffin. The samples were sectioned at a thickness of 5 µm, deparaffinized with xylene, and rehydrated through a graded ethanol series (100%, 95%, and 75%). The sections were subsequently stained with hematoxylin and eosin (H&E), followed by dehydration in 70%, 95%, and 100% ethanol, and cleared with xylene. All slides were examined and imaged using a light microscope equipped with a digital camera system (Olympus, Tokyo, Japan).

For morphometric analysis, transverse H&E-stained sections of the small intestinal mucosa were evaluated under the microscope. Only images captured at 100x magnification were used for measurements of villous height (VH) and crypt depth (CD). Ten villi and crypts were randomly selected per section, and the mean VH and CD values were calculated for each slide. These values were used for statistical comparison between the groups and for determining villous height-to-crypt depth (VH/CD) ratios.

### 2.3. Immunohistochemistry

Paraffin-embedded tissue sections were deparaffinized in xylene and rehydrated through a graded ethanol series. Antigen retrieval was performed by immersing the slides in Tris–EDTA buffer (pH 9.0) at 95 °C for 30 min, followed by three washes with 0.1% Tween 20 in Tris-buffered saline (TBST). To block nonspecific protein binding, the slides were incubated with 5% normal goat serum in TBST at room temperature (25 °C) for 30 min. The sections were then incubated overnight at 4 °C with a 1:200 (*v*/*v*) dilution of anti-intestinal alkaline phosphatase antibody (ab186422, Abcam, UK). Endogenous peroxidase activity was quenched using 3% hydrogen peroxide in TBS. After three washes with 0.1% TBST, the slides were incubated with a 1:1000 (*v*/*v*) dilution of horseradish peroxidase (HRP)-conjugated goat anti-rabbit IgG (KPL, Gaithersburg, MD, USA) for 30 min at room temperature (25 °C). Immunoreactivity was visualized using a DAB Detection Kit (Vector Laboratories, Newark, CA, USA), and the nuclei were counterstained with hematoxylin. Negative controls were performed by omitting the primary antibody, and healthy pig tissues were used as positive controls to confirm antibody specificity. The slides were dehydrated in 70%, 95%, and 100% ethanol, cleared in xylene, and mounted. All samples were observed and imaged using a light microscope equipped with a digital camera system (Olympus, Tokyo, Japan). The intensity of IAP expression was analyzed using Fiji (ImageJ version 1.53t; NIH, Bethesda, MD, USA).

### 2.4. Western Blotting

Intestinal tissues were lysed in RIPA buffer (50 mM Tris-HCl, pH 7.4; 150 mM NaCl; 0.25% sodium deoxycholate; 1% Triton X-100; and 1 mM EDTA), supplemented with 1% protease inhibitor cocktail (Sigma-Aldrich, St. Louis, MO, USA). The lysates were centrifuged at 15,000× *g* for 20 min at 4 °C, and the supernatant was collected. Total protein concentrations were determined using the Bradford assay (Bio-Rad, Hercules, CA, USA) with bovine serum albumin as the standard. Equal amounts of protein (10 µg per sample) were mixed with 2× loading buffer and denatured at 95 °C for 5 min.

Proteins were separated using 10% SDS–PAGE and transferred to polyvinylidene fluoride (PVDF) membranes (Bio-Rad, Hercules, CA, USA) under electrophoresis conditions (200 V or 20 mA). The membranes were blocked in TBS (50 mM Tris-HCl, pH 8.0; 150 mM NaCl; 0.1%) containing 5% bovine serum albumin (BSA) for 1 h at room temperature (25 °C). The blots were incubated overnight at 4 °C with a 1:2000 dilution of anti-intestinal alkaline phosphatase (IAP) antibody (Abcam, Cambridge, UK). The following day, the membranes were washed five times (5 min each) in TBST (TBS with 0.1% Tween-20) and incubated for 1 h at 25 °C with horseradish peroxidase (HRP)-conjugated goat anti-rabbit IgG secondary antibody (Sigma, USA) at a 1:10,000 dilution. After additional washing in TBST and a final rinse in TBS, the signal was developed using Clarity™ Western ECL Substrate (Bio-Rad, Hercules, CA, USA) and visualized using a ChemiDoc Imaging System (Bio-Rad, USA). Band intensities were quantified using Image Lab software (Bio-Rad, Hercules, CA, USA) and were expressed as relative optical density.

To detect the internal control protein, the membranes were stripped with stripping buffer (10% SDS, 0.5 M Tris-HCl, pH 6.8, and 2-mercaptoethanol) at 50 °C for 45 min. The membranes were then washed with TBST, blocked again with 5% BSA in TBS, and reprobed with anti-β-actin antibody (Abcam, Cambridge, UK) at a 1:5000 dilution. HRP-conjugated goat anti-mouse IgG (Sigma, St. Louis, MO, USA) was used as the secondary antibody. The remaining steps were performed as described for IAP detection.

### 2.5. Real-Time Polymerase Chain Reaction (qPCR)

Total RNA was extracted from the intestinal samples (duodenum, jejunum, and ileum) using an RNA extraction kit (Geneaid^®^, New Taipei City, Taiwan), according to the manufacturer’s instructions. The concentration and purity of the extracted RNA were determined by measuring the absorbance ratio at 260/280 using a NanoDrop ND1000 spectrophotometer (Thermo Scientific, Wilmington, DE, USA). Complementary DNA (cDNA) was synthesized from total RNA using the Omniscript RT Kit (Qiagen, Hilden, Germany), following the manufacturer’s protocol.

To validate the target gene, selected RNA samples were reverse transcribed into cDNA, followed by gel electrophoresis and gel purification. The purified product was subjected to Sanger sequencing to confirm the identity of the intestinal alkaline phosphatase (IAP) gene. The validated sequence was used to design specific primers for quantitative PCR. The primer sequences (5′–3′) were as follows:

IAP forward: CTAAAGGGGCAGATGAATGG

IAP reverse: CACCTGTCTGTCCACGTTGT

RPL4 forward: CAAGAGTAACTACAACCTTC

RPL4 reverse: GAACTCTACGATGAATCTTC

The qPCR was performed using the following thermal cycling conditions: initial denaturation at 95 °C for 2 min, followed by 40 cycles of denaturation at 95 °C for 30 s and annealing/extension at 72 °C for 1 min.

### 2.6. Statistical Analysis

Statistical analyses were performed using R software version 4.0.2 (R Foundation for Statistical Computing, Vienna, Austria). Student’s *t*-test was used to compare villous height (VH), crypt depth (CD), the villous height-to-crypt depth (VH/CD) ratio, fecal scores, intestinal alkaline phosphatase (IAP) protein expression (assessed by Western blot and DAB staining), and IAP mRNA expression levels between normal piglets and those challenged with *Escherichia coli* K88. A *p*-value of <0.05 was considered statistically significant.

Western blot data were quantified by measuring the area under the curve of immunoreactive bands using Image Lab software version 6.0.1 (Bio-Rad, USA). DAB staining intensity was analyzed using ImageJ software (NIH, Bethesda, MD, USA). IAP mRNA expression levels were determined using quantitative PCR and were normalized to the reference gene RPL4.

## 3. Results

### 3.1. Assessment of Diarrheal Response Induced by E. coli Infection

Fecal consistency was evaluated daily from Day 0 (D0) to Day 5 (D5) post-challenge in both the negative and positive piglet groups. On D0, all piglets exhibited normal fecal scores (score = 1), indicating no pre-existing symptoms. However, from D2 onwards, the positive group showed a marked increase in fecal scores, with values reaching 3–4 by D4–D5, indicative of moderate to severe diarrhea. In contrast, the negative group maintained consistently low scores throughout the study. A detailed summary of individual fecal scores by treatment group and post-challenge day is provided in Table 1. On Day 0, both groups showed uniformly low scores (1.00 ± 0.00). From Day 1 onward, the *E. coli*-infected group displayed significantly higher scores than the control group (*p* < 0.0001). The mean fecal scores in the infected group were above 3.0 from Day 2 to Day 5, with a peak of 3.75 ± 0.16 on Days 3 and 4, clearly indicating diarrhea. In contrast, the control group maintained consistently low scores throughout the trial. These results confirm the successful induction of diarrhea following *E. coli* challenge and support the validity of the infection model.

### 3.2. Mucosal Morphometry

**Figure 1:**
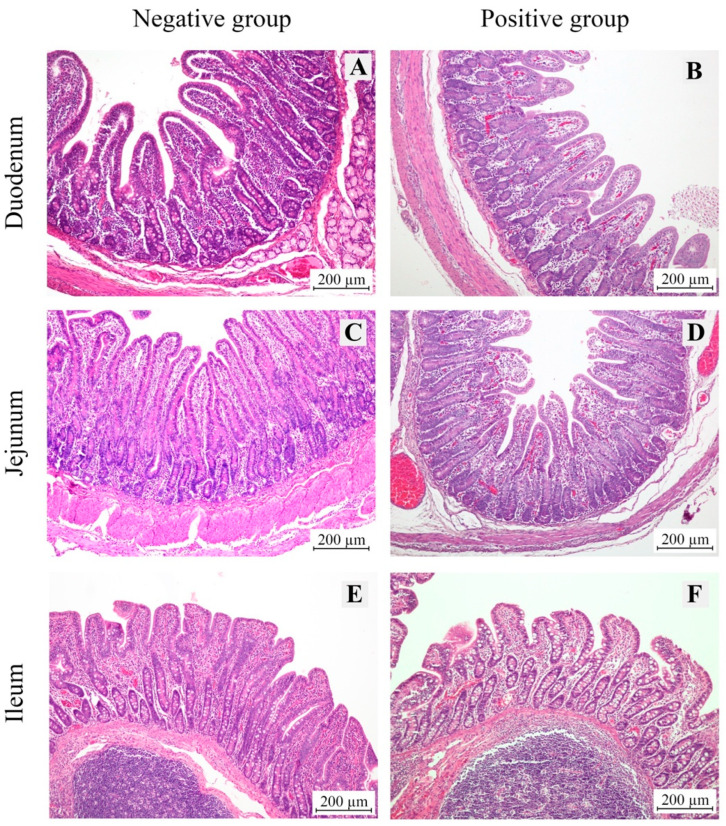
Representative histological sections of the small intestine (duodenum, jejunum, and ileum) from normal pigs (negative group; panels (**A**,**C**,**E**)) and pigs infected with *E. coli* (positive group; panels (**B**,**D**,**F**)). Shortened villi were observed in all segments of the small intestine in the positive group compared to the negative group. All images were captured at 100× magnification. Scale bar = 200 µm.

**Figure 2:**
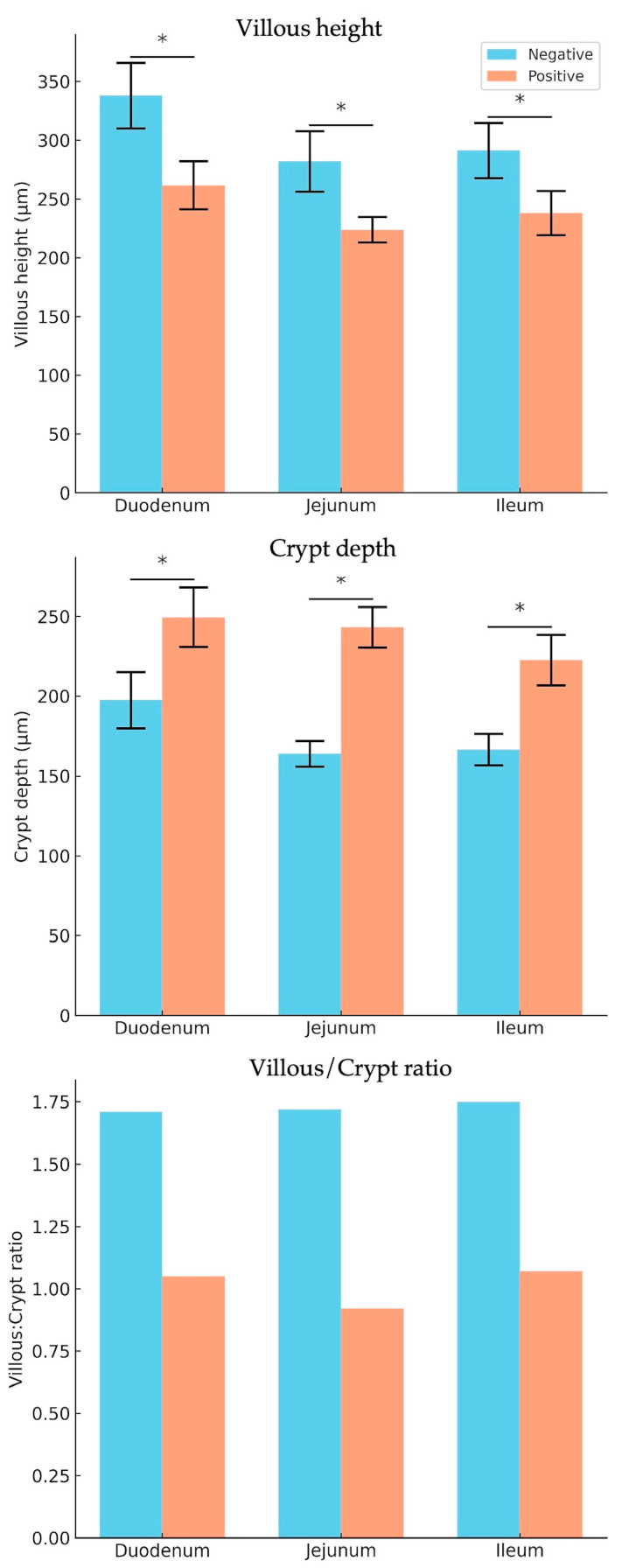
Quantitative analysis of villous height (VH), crypt depth (CD), and villous height-to-crypt depth ratio (VH/CD) in the duodenum, jejunum, and ileum of pigs infected with *E. coli* (positive group) compared to healthy controls (negative group). Data are presented as mean ± standard error of the mean (SEM). Asterisks (*) denote statistically significant differences between groups (*p* < 0.05).

In pigs infected with *E. coli*, the villi appeared shortened, indicating atrophy due to epithelial damage, and exhibited increased degradation at the tips, accompanied by deeper crypts extending into the lamina propria (Figure 1). Morphometric analysis of the villi in the three sections of the small intestine (duodenum, jejunum, and ileum) revealed a significant reduction in villous height in *E. coli*-infected piglets across all three sections compared to the uninfected control group. This reduction in villous height (VH) was statistically significant, as shown in Table 2 and Figure 2. Additionally, this finding was consistent with a significant increase in the depth of the crypts of Lieberkühn in the *E. coli*-infected group. Consequently, the villous height-to-crypt depth ratio (VH/CD ratio) was higher in the control group than in *E. coli*-infected piglets.

### 3.3. Immunohistochemical Analysis of Intestinal Alkaline Phosphatase Expression

**Figure 3:**
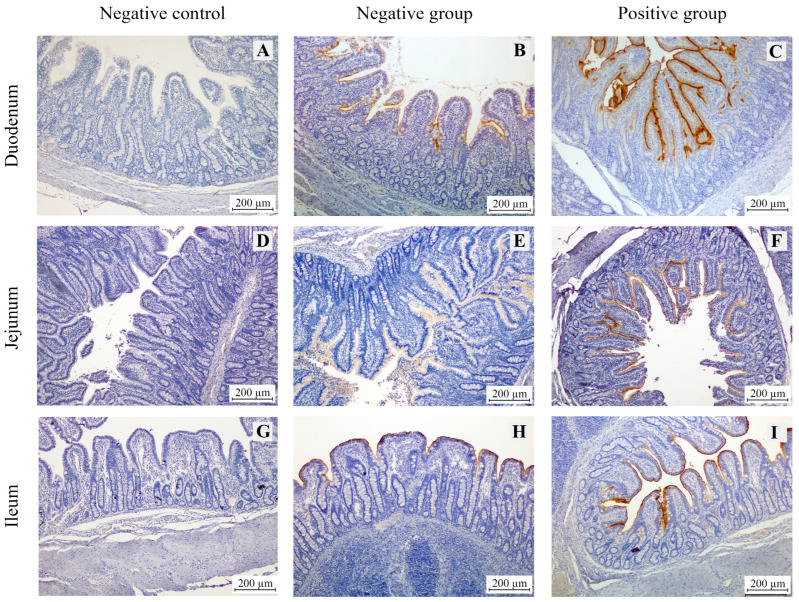
Immunohistochemical staining of intestinal alkaline phosphatase (IAP) in the small intestine of pigs. Panels (**A**,**D**,**G**) show sections from the duodenum, jejunum, and ileum, respectively, in the negative control group (sections processed without the primary antibody). Panels (**B**,**E**,**H**) represent sections from the negative group (healthy pigs), whereas panels (**C**,**F**,**I**) show corresponding sections from the positive group infected with *Escherichia coli*. IAP expression is indicated by brown staining, localized to the apical membrane of villous enterocytes. All images were captured at 100× magnification. Scale bar = 200 µm.

**Figure 4:**
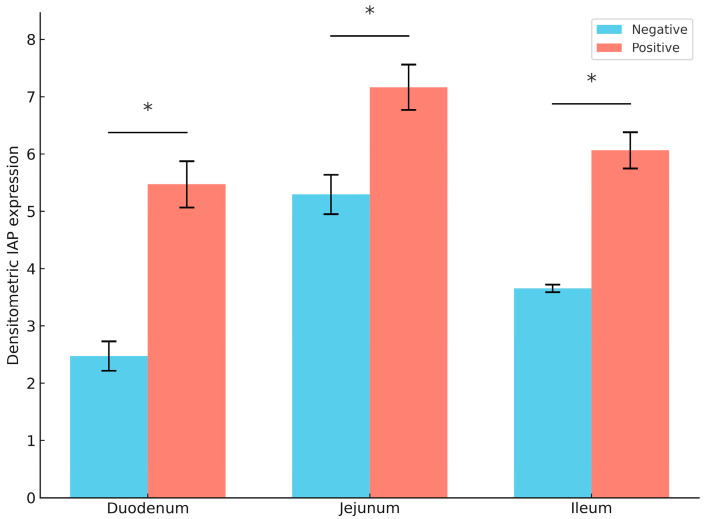
IAP expression in the duodenum, jejunum, and ileum of pigs. The positive group (*E. coli*-infected) exhibited significantly higher expression compared to the negative group (healthy controls). Data are presented as mean ± SEM. Asterisks (*) indicate statistically significant differences (*p* < 0.05).

The immunohistochemical staining results for intestinal alkaline phosphatase (IAP) in the duodenum, jejunum, and ileum of piglets revealed that IAP expression was localized at the tips of the villi in all sections, as indicated by brown staining (Figure 3). Image analysis using ImageJ software showed that the *E. coli*-infected group exhibited significantly higher IAP density in all sections of the small intestine compared to the non-infected group (Figure 4). Among the analyzed intestinal sections, the jejunum exhibited the highest IAP density in both *E. coli*-infected and control piglets (Figure 4).

### 3.4. Western Blot Analysis of Intestinal Alkaline Phosphatase Expression

**Figure 5:**
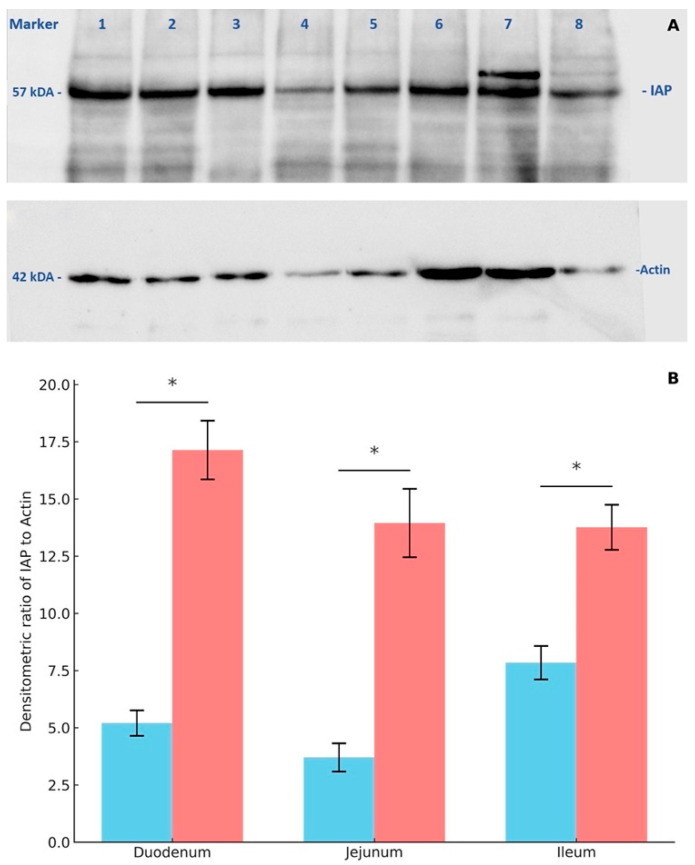
Western blot analysis of IAP expression in small intestinal tissues. (**A**) Representative blots of IAP (~57 kDa) and β-actin (~42 kDa) expression in samples from individual pigs (lanes 1–8). (**B**) Quantitative densitometric analysis of IAP levels normalized to β-actin, showing significantly higher expression in the jejunum and ileum of *E. coli*-infected pigs (positive group) compared to healthy controls (negative group). Data are presented as mean ± SEM. Asterisks (*) indicate statistically significant differences (*p* < 0.05).

The expression of intestinal alkaline phosphatase (IAP) in small intestinal tissues was analyzed through protein extraction and Western blotting, revealing an IAP molecular weight of 57 kDa and a β-actin molecular weight of 42 kDa (Figure 5A). Image analysis using Chemidoc software (version 6.1; Bio-Rad, Hercules, CA, USA) was performed to determine the relative density of the IAP-to-β-actin ratio. The results demonstrate that IAP expression was significantly higher in all sections of the small intestine in *E. coli*-infected piglets compared to the non-infected group (Figure 5B). Notably, IAP expression was significantly elevated in the jejunum and ileum of the *E. coli*-infected group compared to the non-infected group.

### 3.5. Quantitative Real-Time RT-PCR

**Figure 6:**
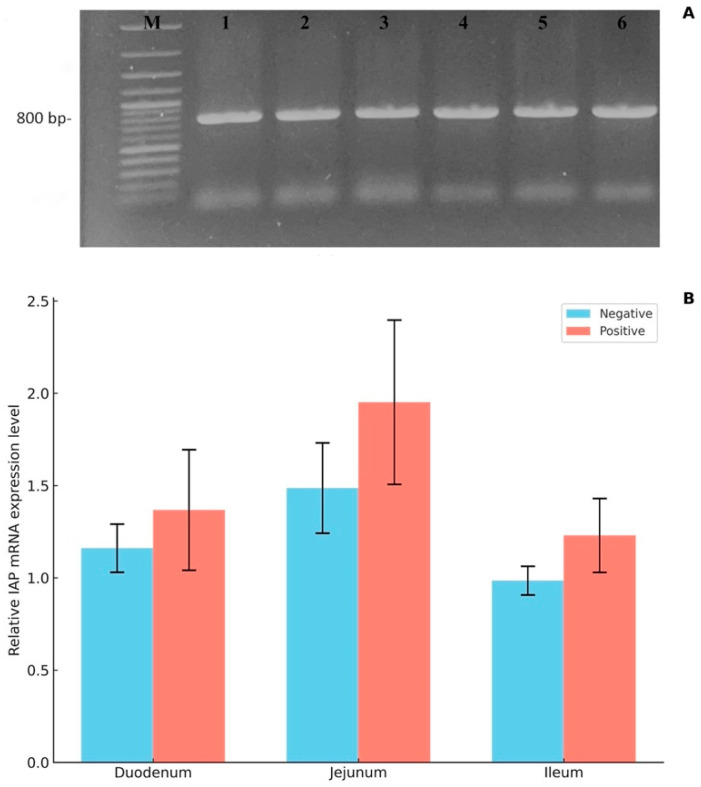
Expression of IAP mRNA in the small intestine of *E. coli*-infected pigs (positive group) and healthy controls (negative group). (**A**) Representative gel electrophoresis showing IAP amplicons (~800 bp) from jejunal samples. (**B**) Quantitative analysis of IAP mRNA expression normalized to RPL4 in the duodenum, jejunum, and ileum showed no statistically significant differences between the groups. Data are presented as mean ± standard error of the mean (SEM), with significance defined as *p* < 0.05.

The mRNA expression of intestinal alkaline phosphatase (IAP) in the small intestine (mid-part of the jejunum) of *E. coli*-infected piglets was analyzed using qPCR with Sybr green and was compared to the non-infected group. Bands corresponding to IAP mRNA expression were detected in all samples, with a band density observed at 800 bp (Figure 6A). The relative expression of IAP was conducted and normalized to RPL4 mRNA expression as an internal control. The results reveal that all segments of the small intestine exhibited higher IAP mRNA expression in *E. coli*-infected piglets compared to the corresponding sections in the non-infected group; however, this difference was not statistically significant (Figure 6B).

## 4. Discussion

In this study, we analyzed the expression of intestinal alkaline phosphatase (IAP) in pigs following an enterotoxigenic *Escherichia coli* (ETEC) K88 challenge and compared this to healthy controls. Post-weaning piglets are highly susceptible to *E. coli* infections, which often result in post-weaning diarrhea (PWD). A survey by Luppi et al. across multiple farms in various European countries identified ETEC as the primary causative agent of PWD, with F4 and F8 ETEC being the most prevalent strains [8].

IAP expression was predominantly observed on the apical surface of villi, consistent with the localization of zebrafish IAP on the intestinal lumen brush border [17]. Notably, IAP expression levels were highest in the jejunum compared to the duodenum and ileum in both healthy and *E. coli*-challenged pigs. This regional variation may be attributed to the higher affinity of membrane-bound IAP in the jejunum, underscoring site-specific differences in IAP distribution along the intestine [18]. Moreover, pigs challenged with *E. coli* exhibited a significant increase in IAP expression across all segments of the small intestine compared to their healthy counterparts, suggesting the potential role of IAP in the intestinal response to *E. coli* infection. Moreover, pigs challenged with E. coli exhibited a significant increase in IAP expression across all segments of the small intestine compared to their healthy counterparts, suggesting the potential role of IAP in the intestinal response to *E. coli* infection.

Although IAP protein expression was markedly increased in the intestinal tissues of *E. coli*-infected pigs, the corresponding mRNA levels did not differ significantly from those in the healthy group. This discrepancy suggests that IAP expression may be regulated at the post-transcriptional or post-translational level. Supporting this notion, Bates et al. [17] demonstrated that IAP activity was induced in zebrafish during gut microbiota colonization without a corresponding increase in mRNA expression, implying transcription-independent activation of the enzyme. Possible mechanisms may include increased protein stability, enzyme phosphorylation, or enhanced catalytic efficiency in response to microbial or inflammatory stimuli [17].

ETEC-induced diarrhea in weaned pigs leads to mucosal inflammation and villous atrophy, as evidenced by a reduction in villous height observed in the duodenum, jejunum, and ileum. These findings align with previous studies [2,4,19]. A decreased villous height-to-crypt depth (VH/CD) ratio was also observed in the small intestine, consistent with previous studies reporting similar findings in various intestinal regions [4,20,21,22]. Given the critical role of mucosal morphometry in the small intestine, particularly the VH/CD ratio, this parameter is fundamental to nutrient absorption [23]. Therefore, it serves as a key indicator in studies investigating intestinal morphology and function.

Lipopolysaccharide (LPS) is a major component of the outer membrane of Gram-negative bacteria, including *E. coli*, and functions as a potent endotoxin by excessively stimulating innate immune signaling through Toll-like receptor 4 (TLR-4) [24]. Upregulation of TLR-4 expression triggers the activation of NF-κB and MAPK pathways, leading to the production of proinflammatory cytokines [7]. Pigs challenged with ETEC or LPS exhibit a pronounced pro-inflammatory response, characterized by increased expression of IL-1β, IL-6, IL-8, IL-17, IL-18, and TNF-α [25,26], while simultaneously suppressing anti-inflammatory cytokines such as IL-4, IL-10 and TGF-β [20].

Intestinal alkaline phosphatase (IAP) plays a crucial role in dephosphorylating LPS, a key component of Gram-negative bacteria [11]. By detoxifying LPS, IAP helps mitigate inflammation, as demonstrated in previous studies [11,15,16,27]. Chen et al. reported that IAP-knockout (IAP-KO) mice challenged with LPS exhibited significantly higher IL-6 levels compared to wild-type (WT) mice [14]. Additionally, dietary supplementation with exogenous IAP has been shown to reduce the incidence of diarrhea, thereby improving growth performance [28,29]. Furthermore, exogenous IAP has been found to support gut microbial homeostasis by promoting the growth of intestinal commensal bacteria while simultaneously inhibiting the Enterobacteriaceae population [29,30,31].

Although our study demonstrates increased IAP expression following *E. coli* infection, we did not evaluate TLR4 expression, proinflammatory cytokines (e.g., IL-6 and TNF-α), or NF-κB activation. Therefore, it remains uncertain whether the elevated IAP levels directly suppress TLR4 signaling in piglets. Nonetheless, based on previous studies in mice demonstrating IAP-mediated inhibition of TLR4 signaling [14,27], it is plausible that IAP may modulate inflammatory responses through a similar pathway in pigs. Further studies are needed to confirm whether this pathway operates similarly in pigs.

## 5. Conclusions

Gram-negative bacteria, such as *Escherichia coli*, can induce intestinal alkaline phosphatase (IAP) expression in response to lipopolysaccharide (LPS), which serves as a substrate and may enhance enzymatic activity. This observation is consistent with the findings of Melo et al., who reported IAP upregulation in the jejunum of pigs challenged with LPS [27]. The upregulation of IAP likely represents an adaptive protective mechanism to detoxify LPS and maintain intestinal homeostasis. These findings highlight the critical role of IAP in promoting gut health, modulating immune responses, and supporting the performance of nursery pigs.

## Figures and Tables

**Table 1:** Fecal scores of individual piglets in the negative and positive groups from Day 0 (D0) to Day 5 (D5) post-challenge.

Group	Animal ID	Fecal Score (Day Post-Challenge)					
		D0	D1	D2	D3	D4	D5
Negative	N1	1	1	1	1	1	1
	N2	1	1	2	2	2	1
	N3	1	1	2	1	2	1
	N4	1	1	1	2	1	1
	N5	1	1	1	1	1	1
	N6	1	1	2	2	1	1
	N7	1	1	1	2	2	1
	N8	1	1	1	1	1	1
Mean ± SEM		1.00 ± 0.00	1.00 ± 0.00	1.38 ± 0.18	1.50 ± 0.19	1.38 ± 0.18	1.00 ± 0.00
Positive	P1	1	2	3	3	4	4
	P2	1	2	4	4	3	3
	P3	1	2	3	4	4	4
	P4	1	2	4	4	3	3
	P5	1	3	3	4	4	4
	P6	1	2	3	3	4	4
	P7	1	2	3	4	4	4
	P8	1	3	4	4	4	3
Mean ± SEM		1.00 ± 0.00	2.25 ± 0.16	3.38 ± 0.18	3.75 ± 0.16	3.75 ± 0.16	3.62 ± 0.18
*p*-value		N/A	<0.0001	<0.0001	<0.0001	<0.0001	<0.0001

N/A: Not applicable.

**Table 2:** Comparison of villous height (VH), crypt depth (CD), and VH/CD ratio in the small intestine of normal and *E. coli* K88-infected piglets. Data are presented as mean ± SEM.

	Group		
**Item**	Negative (n = 8)	Positive (n = 8)	*p*-value
**Duodenum** Villous height (µm)	338.02 ± 27.91	261.53 ± 20.41	0.002
Crypt depth (µm)	197.56 ± 17.57	249.45 ± 18.66	0.001
VH/CD ratio	1.71:1	1.05:1	
**Jejunum** Villous height (µm)	282.00 ± 25.78	223.84 ± 10.77	<0.001
Crypt depth (µm)	163.84 ± 8.10	243.09 ± 12.69	0.002
VH/CD ratio	1.72:1	0.92:1	
**Ileum** Villous height (µm)	291.33 ± 23.48	238.06 ± 18.75	<0.001
Crypt depth (µm)	166.55 ± 9.91	222.56 ± 15.82	<0.001
VH/CD ratio	1.75:1	1.07:1	

## Data Availability

The data presented in this study are available in the article. Further inquiries can be directed to the corresponding author.

## Abbreviations

The following abbreviations are used in this manuscript:

IAP	Intestinal alkaline phosphatase
LPS	Lipopolysaccharide
PWD	Post-weaning diarrhea
ETEC	Enterotoxigenic *Escherichia coli* (ETEC)

## References

[1] Sargeant H.R., McDowall K.J., Miller H.M., Shaw M.A. (2010). Dietary zinc oxide affects the expression of genes associated with inflammation: Transcriptome analysis in piglets challenged with ETEC K88. Vet. Immunol. Immunopathol..

[2] McLamb B.L., Gibson A.J., Overman E.L., Stahl C., Moeser A.J. (2013). Early Weaning Stress in Pigs Impairs Innate Mucosal Immune Responses to Enterotoxigenic *E. coli* Challenge and Exacerbates Intestinal Injury and Clinical Disease. PLoS ONE.

[3] Lu X., Zhang M., Zhao L., Ge K., Wang Z., Jun L., Ren F. (2018). Growth performance and post-weaning diarrhea in piglets fed a diet supplemented with probiotic complexes. J. Microbiol. Biotechnol..

[4] Zou C., Zhao W., Yin S., Xiang X., Tang J., Jia G., Che L., Liu G., Chen X., Tian G. (2024). Artificial parasin I protein (API) supplementation improves growth performance and intestinal health in weaned piglets challenged with enterotoxigenic *Escherichia coli*. Anim. Nutr..

[5] Jenkins T.P., Ács N., Arendrup E.W., Swift A., Duzs A., Chatzigiannidou I., Pichler M., Kittilä T., Peachey L., Gram L. (2024). Protecting the piglet gut microbiota against ETEC-mediated post-weaning diarrhoea using specific binding proteins. npj Biofilms Microbiomes.

[6] Luise D., Lauridsen C., Bosi P., Trevisi P. (2019). Methodology and application of *Escherichia coli* F4 and F18 encoding infection models in post-weaning pigs. J. Anim. Sci. Biotechnol..

[7] Ren W., Yin J., Duan J., Liu G., Zhu X., Chen S., Li T., Wang S., Tang Y., Hardwidge P.R. (2014). Mouse intestinal innate immune responses altered by enterotoxigenic *Escherichia coli* (ETEC) infection. Microbes Infect..

[8] Luppi A., Gibellini M., Gin T., Vangroenweghe F., Vandenbroucke V., Bauerfeind R., Bonilauri P., Labarque G., Hidalgo A. (2016). Prevalence of virulence factors in enterotoxigenic *Escherichia coli* isolated from pigs with post-weaning diarrhoea in Europe. Porc. Health Manag..

[9] Sun Y., Kim S.W. (2017). Intestinal challenge with enterotoxigenic *Escherichia coli* in pigs, and nutritional intervention to prevent postweaning diarrhea. Anim. Nutr..

[10] Estaki M., DeCoffe D., Gibson D.L. (2014). Interplay between intestinal alkaline phosphatase, diet, gut microbes and immunity. World J. Gastroenterol..

[11] Chen K.T., Malo M.S., Moss A.K., Zeller S., Johnson P., Ebrahimi F., Mostafa G., Alam S.N., Ramasamy S., Warren H.S. (2010). Identification of specific targets for the gut mucosal defense factor intestinal alkaline phosphatase. Am. J. Physiol. Gastrointest. Liver Physiol..

[12] Fawley J., Gourlay D.M. (2016). Intestinal alkaline phosphatase: A summary of its role in clinical disease. J. Surg. Res..

[13] Molnár K., Vannay A., Szebeni B., Bánki N.F., Sziksz E., Cseh A., Győrffy H., Lakatos P.L., Papp M., Arató A. (2012). Intestinal alkaline phosphatase in the colonic mucosa of children with inflammatory bowel disease. World J. Gastroenterol..

[14] Chen K.T., Malo M.S., Beasley-Topliffe L.K., Poelstra K., Millan J.L., Mostafa G., Alam S.N., Ramasamy S., Warren H.S., Hohmann E.L. (2011). A role for intestinal alkaline phosphatase in the maintenance of local gut immunity. Dig. Dis. Sci..

[15] Koyama I., Matsunaga T., Harada T., Hokari S., Komoda T. (2002). Alkaline phosphatases reduce toxicity of lipopolysaccharides in vivo and in vitro through dephosphorylation. Clin. Biochem..

[16] Beumer C., Wulferink M., Raaben W., Fiechter D., Brands R., Seinen W. (2003). Calf Intestinal Alkaline Phosphatase, a Novel Therapeutic Drug for Lipopolysaccharide (LPS)-Mediated Diseases, Attenuates LPS Toxicity in Mice and Piglets. J. Pharmacol. Exp. Ther..

[17] Bates J.M., Akerlund J., Mittge E., Guillemin K. (2007). Intestinal Alkaline Phosphatase Detoxifies Lipopolysaccharide and Prevents Inflammation in Zebrafish in Response to the Gut Microbiota. Cell Host Microbe.

[18] Fan M.Z., Adeola O., Asem E.K. (1999). Characterization of brush border membrane-bound alkaline phosphatase activity in different segments of the porcine small intestine. J. Nutr. Biochem..

[19] Peng S.S., Li Y., Chen Q., Hu Q., He Y., Che L., Jiang P.P. (2022). Intestinal and mucosal microbiome response to oral challenge of enterotoxigenic *Escherichia coli* in weaned Pigs. Pathogens.

[20] Gao Y., Han F., Rong Y., Yi H., Wang Y. (2013). Changes in gut microbial populations, intestinal morphology, expression of tight junction proteins, and cytokine production between two pig breeds after challenge with *Escherichia coli* K88: A comparative study. J. Anim. Sci..

[21] Zhang K., Shen X., Han L., Wang M., Lian S., Wang K., Li C. (2023). Effects on the intestinal morphology, inflammatory response and microflora in piglets challenged with enterotoxigenic *Escherichia coli* K88. Res. Vet. Sci..

[22] Yi Q., Liu J., Zhang Y., Qiao H., Chen F., Zhang S., Guan W. (2021). Anethole Attenuates Enterotoxigenic *Escherichia coli*-Induced Intestinal Barrier Disruption and Intestinal Inflammation via Modification of TLR Signaling and Intestinal Microbiota. Front. Microbiol..

[23] Nguyen D.T.N., Le N.H., Pham V.V., Eva P., Alberto F., Le H.T. (2021). Relationship between the ratio of villous height:crypt depth and gut bacteria counts as well production parameters in broiler chickens. J. Agric. Dev..

[24] Santos G.M., Ismael S., Morais J., Araújo J.R., Faria A., Calhau C., Marques C. (2022). Intestinal Alkaline Phosphatase: A Review of This Enzyme Role in the Intestinal Barrier Function. Microorganisms.

[25] Vermeire B., Walsh M., Cox E., Devriendt B. (2024). The lipopolysaccharide structure affects the detoxifying ability of intestinal alkaline phosphatases. BMC Vet. Res..

[26] Ren W., Yin J., Chen S., Duan J., Liu G., Li T., Li N., Peng Y., Tan B., Yin Y. (2016). Proteome analysis for the global proteins in the jejunum tissues of enterotoxigenic *Escherichia coli*-infected piglets. Sci. Rep..

[27] Melo A.D.B., Silveira H., Bortoluzzi C., Lara L.J., Garbossa C.A.P., Preis G., Costa L.B., Rostagno M.H. (2016). Intestinal alkaline phosphatase and sodium butyrate may be beneficial in attenuating LPS-induced intestinal inflammation. Genet. Mol. Res..

[28] Genova L.J., Rupolo P.E., Melo A.D.B., Santos L.B.A., Wendt G.N., Barbosa K.A., Carvalho S.T., Oliveira N.T.E., Costa L.B., Carvalho P.L.O. (2021). Biological response of piglets challenged with *Escherichia coli* F4 (K88) when fed diets containing intestinal alkaline phosphatase. Czech J. Anim. Sci..

[29] Genova J.L., Melo A.D.B., Rupolo P.E., Macedo R.E.F., Filho J.R.E., Carvalho S.T., Faucitano L., Costa L.B., Carvalho P.L.O. (2022). New findings of intestinal alkaline phosphatase: Effects on intestinal and organ health of piglets challenged with ETEC F4 (K88). Rev. Bras. Zootec..

[30] Malo M.S., Moaven O., Muhammad N., Biswas B., Alam S.N., Economopoulos K.P., Gul S.S., Hamarneh S.R., Malo N.S., Teshager A. (2014). Intestinal alkaline phosphatase promotes gut bacterial growth by reducing the concentration of luminal nucleotide triphosphates. Am. J. Physiol. Gastrointest. Liver Physiol..

[31] Malo M.S., Alam S.N., Mostafa G., Zeller S.J., Johnson P.V., Mohammad N., Chen K.T., Moss A.K., Ramasamy S., Faruqui A. (2010). Intestinal alkaline phosphatase preserves the normal homeostasis of gut microbiota. Gut.

